# Psychiatric Comorbidities and Schizophrenia in Youths With Attention-Deficit/Hyperactivity Disorder

**DOI:** 10.1001/jamanetworkopen.2023.45793

**Published:** 2023-11-30

**Authors:** Soo Min Jeon, Dong Yun Lee, SangHun Cha, Jin-Won Kwon

**Affiliations:** 1 College of Pharmacy, Jeju National University, Jeju, South Korea; 2 Department of Biomedical Informatics, Ajou University School of Medicine, Suwon, South Korea; 3 Department of Statistics, College of Natural Sciences, Kyungpook National University, Daegu, South Korea; 4 BK21 FOUR Community-Based Intelligent Novel Drug Discovery Education Unit, College of Pharmacy and Research Institute of Pharmaceutical Sciences, Kyungpook National University, Daegu, South Korea

## Abstract

**Importance:**

The association between attention-deficit/hyperactivity disorder (ADHD) and schizophrenia has received increased attention; however, evidence on the association between psychiatric comorbidities and subsequent schizophrenia in patients with ADHD is limited.

**Objective:**

To investigate the risk of being diagnosed with schizophrenia in children and adolescents with ADHD considering the presence of psychiatric comorbidity.

**Design, Setting, and Participants:**

This was a population-based, retrospective cohort study using the Health Insurance Review and Assessment claims database from January 1, 2007, to December 31, 2019. Participants were children and adolescents aged 5 to 19 years who received an ADHD diagnosis between January 1, 2010, and December 31, 2018, in the nationwide claims data of Korea. Data were analyzed from January 2010 to December 2019.

**Interventions or Exposures:**

The presence of psychiatric comorbidity was assessed from diagnosis records within 1 year before ADHD diagnosis. Comorbidities were further categorized according to the number of comorbidities and specific comorbid disorders.

**Main Outcomes and Measures:**

Cox proportional hazard models were used to estimate hazard ratios (HRs) and 95% CIs, examining the association between psychiatric comorbidities and the risk of being diagnosed with schizophrenia. Furthermore, the occurrence of psychiatric comorbidity during the follow-up period was explored among patients without psychiatric comorbidity at baseline.

**Results:**

A total of 211 705 patients with newly diagnosed ADHD were included. A total of 157 272 patients (74.3%) were male, and the age of 5 to 9 years showed the highest distribution (115 081 patients [54.4%]). Patients with psychiatric comorbidity had a significantly higher risk of being diagnosed with schizophrenia than those without (adjusted HR, 2.14; 95% CI, 2.05-2.23). The association between schizophrenia and psychiatric comorbidity became progressively greater with the increasing number of comorbidities. Several individual psychiatric disorders showed an association with development of schizophrenia, with ASD, intellectual disability, tic disorder, depression, and bipolar disorder being the top 5 disorders most associated. Furthermore, 3244 patients (73.8%) without psychiatric comorbidities experienced the emergence of other psychiatric disorders before schizophrenia occurrence.

**Conclusions and Relevance:**

In this retrospective cohort study involving children and adolescents with ADHD, the presence of psychiatric comorbidity in patients with ADHD was associated with an increased risk of being diagnosed with schizophrenia, with an increased risk observed in multiple comorbidities and a wide variety of comorbidities. These findings highlight the significance of assessing and managing psychiatric comorbidities in patients with ADHD to decrease subsequent schizophrenia risk and allow for early intervention.

## Key Points

**Question:**

Are psychiatric comorbidities in youths with attention-deficit/hyperactivity disorder (ADHD) who are followed up longitudinally associated with higher rates of schizophrenia?

**Findings:**

In this cohort study involving 211 705 children and adolescents with ADHD, patients with psychiatric comorbidities had an approximately 2.1-fold higher risk of being diagnosed with schizophrenia compared with those without comorbidities. The higher risk of subsequent schizophrenia was associated with both a greater number of comorbidities and a variety of individual psychiatric disorders.

**Meaning:**

These findings suggest that many people with ADHD who receive schizophrenia diagnoses have various psychiatric comorbidities at the time of diagnosis and experience various disorders preceding the diagnosis.

## Teaser

This cohort study evaluates the association between psychiatric comorbidities and schizophrenia in children and adolescents with attention-deficit/hyperactivity disorder.

## Introduction

Attention-deficit/hyperactivity disorder (ADHD) is the most common neurodevelopmental disorder in children and adolescents, characterized by impulsivity, hyperactivity, and poor attention.^1^ There is growing evidence that ADHD is commonly associated with schizophrenia.^2,3,4,5,6^ One previous systematic review and meta-analysis^5^ of observational studies demonstrated that ADHD is statistically significantly associated with the incidence of subsequent schizophrenia, with a relative risk of 4.74 (95% CI, 4.11-5.46). Since schizophrenia negatively influences quality of life, including academic functioning, social functioning, and mortality,^7^ early detection and intervention for schizophrenia for children and adolescents with ADHD have long been considered important.^5^

To control the risk of developing subsequent schizophrenia among patients with ADHD, understanding the mechanisms and risk factors associated with these 2 disorders is essential. Several previous studies explored the relationship between ADHD and schizophrenia regarding environmental risk factors^8^ and shared genetic susceptibility.^9,10,11^ However, studies investigating the influence of psychiatric comorbidity on diagnosis of schizophrenia in patients with ADHD are limited. Psychiatric comorbidity is commonly observed in patients with ADHD.^12,13^ Although the prevalence rate was varied, approximately 50% to 90% of patients with ADHD have at least 1 other psychiatric comorbid disorder, including depression, bipolar disorder, anxiety, and autism spectrum disorder (ASD).^14,15,16,17,18^ The presence of psychiatric comorbidity in patients with ADHD has significant implications for symptom severity, functional impairment, and treatment outcomes.^13^ Furthermore, these psychiatric disorders were independently associated with an increased risk of schizophrenia.^19,20,21,22^ Moreover, most patients exhibited several clinical symptoms of other psychiatric disorders before schizophrenia occurrence. Therefore, the notion that the presence of psychiatric comorbidity may further be associated with being diagnosed with schizophrenia in this population is plausible. Thus, we aimed to explore the association between ADHD and subsequent schizophrenia and patterns of diagnosis of schizophrenia in children and adolescents with ADHD, focusing on comorbidity.

## Methods

### Data Sources

We conducted a nationwide population-based retrospective cohort study using the Health Insurance Review and Assessment Service (HIRA) database of South Korea (from January 1, 2007, to December 31, 2019). HIRA is a government-operated agency that evaluates the reimbursement system, which is mandatory for all Korean populations. In this study, we used information about pediatric patients with psychiatric disorders from the HIRA database from 2007 to 2019. The HIRA database contains individual characteristics, including age, sex, and economic vulnerability (ie, approximately 2.8% of the total population who qualify for medical insurance advantage beneficiaries). Clinical data include information billed by health care practitioners, including diagnoses (according to the *International Statistical Classification of Diseases and Related Health Problems, Tenth Revision [ICD-10]*), prescriptions, procedures, and devices. This study was approved by the institutional review board of Jeju National University, and the requirement for informed consent was waived as the HIRA database was anonymized. This study adhered to the Strengthening the Reporting of Observational Studies in Epidemiology (STROBE) reporting guideline for cohort study.

### Study Participants

Initially, we identified patients aged 5 to 19 years with at least 1 diagnosis record of ADHD (*ICD-10*: F90) between January 1, 2010, and December 31, 2018. The index date for individual patients was defined as the first date of ADHD diagnosis. We excluded patients with a history of ADHD diagnosis or prescription of stimulants and nonstimulants for ADHD for at least 3 years before the index date to restrict the cohort for new incident cases of ADHD. We subsequently only included those who had no diagnosis record of schizophrenia or psychosis for at least 3 years before the index date. The time frame of 3 years was selected according to previous studies that reported the recovery of schizophrenia or psychosis, measured for at least 2 years after treatment.^23,24,25^ The detailed codes of diagnosis and medications were summarized in eTable 1 in Supplement 1.

All study populations were followed up from the index date to the first diagnosis date of schizophrenia or end of follow-up (December 31, 2019), whichever occurred earlier. Schizophrenia development was defined according to the *ICD-10* code of F2 following ADHD diagnosis.

### Association of Being Diagnosed With Schizophrenia With the Psychiatric Comorbidity of ADHD

We defined depression, anxiety disorder, intellectual disability, nonorganic sleep disorder, tic disorder, bipolar disorder, ASD, conduct disorder, obsessive-compulsive disorder, and substance use disorder (SUD) as psychiatric comorbid disorders of ADHD. The detailed *ICD-10* codes of psychiatric comorbidities are shown in eTable 2 in Supplement 1. These psychiatric comorbid disorders were selected according to previous studies.^12,17,26,27^

In this study, we classified patients into 2 groups according to the presence of psychiatric comorbidity within 1 year before the index date. Patients who had at least 1 diagnosis record of these psychiatric disorders within 1 year before the index date belonged to the group with psychiatric comorbidities, and others were categorized into the group without psychiatric disorders. Among those with underlying psychiatric comorbidities, further categorization was conducted according to the number of psychiatric comorbidities and specific psychiatric disorders. The number of psychiatric comorbidities was divided into the following 3 groups: 1 comorbidity, 2 comorbidities, and 3 or more comorbidities. For each psychiatric disorder, patients who had at least 1 diagnostic record of that specific disorder were considered as having that particular disorder. To simultaneously investigate the outcomes of both multiple comorbidities and a specific disorder, patients were additionally categorized into the following 3 groups: 1 comorbidity for each psychiatric disorder, 2 comorbidities, and 3 or more comorbidities.

To estimate hazard ratios (HRs) along with 95% CIs for the association of being diagnosed with schizophrenia with psychiatric comorbidity in patients with ADHD, Cox proportional hazard models (Cox PH models) were used. We considered several potential confounders in schizophrenia development, including sex, age (categorized as 5-9, 10-14, and 15-19 years), insurance type (health insurance or Medicaid), psychiatric hospitalization, and psychotropic medication use. Age, sex, insurance type, and psychiatric hospitalization were assessed at the index date. The use of psychotropic medications (ie, antipsychotics, antidepressants, anxiolytics, anticholinergics, antiepileptics, and lithium) was assessed during the 1-year period before the index date (eTable 3 in Supplement 1). We used 2 models adjusting different potential confounders. Model 1 adjusted sex, age, insurance type, and psychiatric hospitalization as covariates. Model 2 further adjusted for psychotropic medication use as a covariate.

Moreover, we conducted various sensitivity analyses to examine the robustness of main finings. First, to evaluate whether increasing age is associated with the diagnosis of schizophrenia, we performed a subgroup analysis by age group. Second, we performed a subgroup analysis according to the use of methylphenidate (the only approved stimulant for ADHD in South Korea) during the follow-up period. Additionally, we conducted subgroup analyses according to presence of bipolar disorder, ASD, and intellectual disability at baseline period. Third, to further address uncontrolled confounding effects, we performed an analysis using a propensity score (PS)–matched cohort. We used logistic regression to estimate the PS for the probability of having psychiatric comorbidities by including sex, age, health insurance, and health care utilization (overall visit frequency, psychiatric visit frequency, nonpsychiatric hospital visit frequency). Patients with psychiatric comorbidities and those without were matched 1:1 using the estimated PS with a caliper width of 0.2 on the PS scale.

### Patterns of Psychiatric Comorbid Diagnosis of Schizophrenia Following ADHD

During the follow-up period, individuals with ADHD might experience various psychiatric comorbidities before the subsequent incidence of schizophrenia. However, in the association analysis, it is difficult to capture the comprehensive longitudinal diagnosis pattern from ADHD to schizophrenia due to the complex trajectories of these disorders. To address this, we used trajectory analysis to examine the progression of psychotic disorders to a schizophrenia diagnosis in patients with ADHD.

We assessed the occurrence of psychiatric comorbid disorders during the period between the index date of ADHD and subsequent schizophrenia to explore the patterns of diagnosis from ADHD to schizophrenia. This analysis exclusively focused on patients who received schizophrenia diagnoses without any preexisting psychiatric comorbidities (patients belonging to the group without psychiatric comorbidities), ensuring that the diagnostic progression was not influenced by prior cumulative diagnoses. During the follow-up period, the first date of diagnosis record for each psychiatric disorder was considered the occurrence of psychiatric comorbidities. The investigation of diagnostic patterns from ADHD to schizophrenia was presented as frequency and incidence rate per 100 person-years. When 2 or more psychiatric disorders occurred simultaneously on the same day, we adjusted weight of incidence by dividing the number of psychiatric comorbid disorders. The diagnostic flow was depicted using a Sankey diagram.

### Statistical Analysis

SAS Enterprise Guide 6.1 (SAS Institute) and R software V.3.6.1 (R Project for Statistical Computing) were used for data analyses. The baseline demographics of the patients were presented as numbers and proportions for the 2 groups according to the presence of psychiatric comorbidities. The comparison between these groups was performed using 2-sided χ^2^ tests with significance set at less than .05. Data were analyzed from January 2010 to December 2019.

## Results

We identified a total of 211 705 patients to be involved in our study cohort. The majority were male (157 272 patients [74.3%]), and 115 081 patients (54.4%) were aged 5 to 9 years. Among them, 77 880 (36.8%) had at least 1 diagnosis record of psychiatric disorders within 1 year before the index date, whereas 133 825 (63.2%) did not exhibit any comorbidities (eFigure 1 in Supplement 1; Table 1). Over a mean (SD) follow-up period of 5.57 (2.81) years, 6448 patients (8.28%) with psychiatric comorbidities received schizophrenia diagnoses. Meanwhile, 4396 patients (3.28%) without psychiatric comorbidities received schizophrenia diagnoses during a mean (SD) follow-up of 6.61 (2.71) years. The most common insurance type was health insurance (196 684 patients [92.9%]). A total of 263 patients (0.1%) experienced psychiatric hospitalization within 1 year before the index date. The most used psychotropic medications were anxiolytics (45 730 patients [21.6%]), followed by selective serotonin reuptake inhibitors or serotonin and norepinephrine reuptake inhibitors (SSRIs/SNRIs) (14 861 patients [7.0%]). Patients with psychiatric comorbidities were older, were more likely to be female, and exhibited a greater psychotropic medication use than those without psychiatric comorbidities (these differences were statistically significant). Among diverse psychiatric comorbid disorders, depression (30 091 patients [38.6%]) and anxiety disorder (19 445 patients [25.0%]) were the 2 most common psychiatric disorders.

**Table 1.** Demographics of Patients With Attention-Deficit/Hyperactivity Disorder (ADHD) According to the Presence of Psychiatric Comorbidities

Demographic	Participants, No. (%)			*P* value
	Total patients (N = 211 705)	ADHD with psychiatric comorbidities (n = 77 880)	ADHD without psychiatric comorbidities (n = 133 825)	
Schizophrenia diagnosis	10 844 (5.12)	6448 (8.28)	4396 (3.28)	NA
Follow-up, median (SD), y	6.31 (2.77)	5.57 (2.81)	6.61 (2.71)	NA
Sex				
Male	157 272 (74.29)	53 912 (69.22)	103 360 (77.24)	<.001
Female	54 433 (25.71)	23 968 (30.78)	30 465 (22.76)	
Age, y				
5-9	115 081 (54.36)	31 647 (40.64)	83 434 (62.35)	<.001
10-14	64 591 (30.51)	26 309 (33.78)	38 282 (28.61)	
15-19	32 033 (15.13)	19 924 (25.58)	12 109 (9.05)	
Insurance type				
Health insurance	196 684 (92.90)	71 636 (91.98)	125 048 (93.44)	.006
Medicaid	15 021 (7.10)	6244 (8.02)	8777 (6.56)	
Psychiatric hospitalization^a^	263 (0.12)	243 (0.31)	20 (0.01)	<.001
Psychiatric comorbidities, No.^a^				NA
1	56 593 (26.73)	56 593 (72.67)	NA	NA
2	16 563 (7.82)	16 563 (21.27)	NA	NA
≥3	4724 (2.23)	4724 (6.07)	NA	NA
Individual psychiatric comorbid disorder^b^				
Depression	30 091 (14.21)	30 091 (38.64)	NA	NA
Anxiety disorder	19 445 (9.18)	19 445 (24.97)	NA	NA
Tic disorder	15 460 (7.30)	15 460 (19.85)	NA	NA
Intellectual disability	12 440 (5.88)	12 440 (15.97)	NA	NA
Conduct disorder	10 515 (4.97)	10 515 (13.50)	NA	NA
ASD	6867 (3.24)	6867 (8.82)	NA	NA
Bipolar disorder	4358 (2.06)	4358 (5.60)	NA	NA
OCD	3095 (1.46)	3095 (3.97)	NA	NA
Nonorganic sleep disorder	2446 (1.16)	2446 (3.14)	NA	NA
SUD	318 (0.15)	318 (0.40)	NA	NA
Use of psychotropic medications^a^				
Typical antipsychotics	1648 (0.78)	1593 (2.05)	55 (0.04)	<.001
Atypical antipsychotics	8624 (4.07)	8344 (10.71)	280 (0.21)	<.001
Anticholinergics	2318 (1.09)	2253 (2.89)	65 (0.05)	<.001
Anxiolytics	45 730 (21.60)	19 328 (24.82)	26 402 (19.73)	<.001
SSRIs/SNRIs	14 861 (7.02)	13 311 (17.09)	1550 (1.16)	<.001
TCAs	976 (0.46)	799 (1.03)	177 (0.13)	<.001
MAOIs and others	2208 (1.04)	2016 (2.59)	192 (0.14)	<.001
Antiepileptics	6866 (3.24)	4460 (5.73)	2406 (1.80)	<.001
Lithium	652 (0.31)	608 (0.78)	44 (0.03)	<.001

Abbreviations: ASD, autism spectrum disorder; MAOIs, monoamine oxidase inhibitors; NA, not applicable; OCD, obsessive-compulsive disorder; SNRIs, serotonin and norepinephrine reuptake inhibitors; SSRIs, selective serotonin reuptake inhibitors; SUD, substance use disorder; TCAs, tricyclic antidepressants.

^a^ History of psychiatric hospitalization, psychiatric comorbidities, and the use of psychotropic medications were evaluated within 1 year before the index date.

^b^ Patients with at least 1 diagnosis record of each disorder were considered to have each disorder.

The results of Cox PH regression analysis for the risk of schizophrenia according to the presence of psychiatric comorbidities in patients with ADHD with and without adjustment for other psychotropic medications are shown in Table 2. In both models, the presence of comorbidities was associated with an increased subsequent schizophrenia risk among patients with ADHD (model 1 HR, 2.51; 95% CI, 2.41-2.61; model 2 HR, 2.14; 95% CI, 2.05-2.23). Furthermore, we observed that male sex, older age, and Medicaid were associated with increased schizophrenia risk. Regarding psychotropic medication use, typical antipsychotics, atypical antipsychotics, anxiolytics, anticholinergics, SSRIs/SNRIs, and antiepileptics showed an increased subsequent schizophrenia risk.

**Table 2.** Association Between Being Diagnosed With Schizophrenia and Psychiatric Comorbidities in Patients With Attention-Deficit/Hyperactivity Disorder (ADHD)

Characteristic	HR (95% CI)	
	Model 1^a^	Model 2^b^
Sex (reference, male)	0.93 (0.89-0.97)	0.91 (0.87-0.95)
Age, y (reference, 5-9)		
10-14	1.14 (1.09-1.19)	1.12 (1.07-1.17)
15-19	1.62 (1.55-1.71)	1.47 (1.40-1.55)
Medicaid (reference, health insurance)	1.86 (1.75-1.97)	1.86 (1.75-1.97)
Psychiatric hospitalization (reference, none)	1.73 (1.35-2.21)	1.05 (0.82-1.35)
Psychiatric comorbidity (reference, no psychiatric comorbidities)	2.51 (2.41-2.61)	2.14 (2.05-2.23)
Psychotropic medication use (reference, none)		
Typical antipsychotics	NA	1.35 (1.19-1.53)
Atypical antipsychotics	NA	1.96 (1.82-2.11)
Anticholinergics	NA	1.41 (1.26-1.58)
Anxiolytics	NA	1.10 (1.05-1.15)
SSRIs/SNRIs	NA	1.26 (1.18-1.34)
TCAs	NA	1.11 (0.93-1.33)
MAOIs and others	NA	0.96 (0.84-1.09)
Antiepileptics	NA	1.26 (1.17-1.37)
Lithium	NA	1.16 (0.98-1.39)

Abbreviations: HR, hazard ratio; MAOIs, monoamine oxidase inhibitors; NA, not applicable; SNRIs, serotonin and norepinephrine reuptake inhibitors; SSRIs, selective serotonin reuptake inhibitors; TCAs, tricyclic antidepressants.

^a^ Model 1 adjusted sex, age, health insurance, and psychiatric hospitalization as covariates.

^b^ Model 2 adjusted sex, age, health insurance, psychiatric hospitalization, and psychotropic medication use as covariates.

The association between being diagnosed with schizophrenia and psychiatric comorbidities according to several studies is depicted in Table 3. With the increasing number of psychiatric comorbidities, patients with psychiatric comorbidities had an increased schizophrenia risk compared with those without psychiatric comorbidities. In model 2, the risk of being diagnosed with schizophrenia was approximately 1.91-fold for 1 comorbidity, 2.94-fold for 2 comorbidities, and 4.26-fold higher for 3 or more comorbidities than those without psychiatric comorbidities. Regarding individual psychiatric comorbidities, the increased risk of subsequent schizophrenia was significantly associated with the presence of all individual comorbidities, excluding SUD. Among them, ASD exhibited the greatest association with schizophrenia risk, with HRs of 2.72 (95% CI, 2.52-2.92) and 2.43 (95% CI, 2.26-2.62) in models 1 and 2, respectively. Moreover, intellectual disability, tic disorder, depression, and bipolar disorder showed a remarkable association, with HRs of 1.83 (95% CI, 1.72-1.95), 1.77 (95% CI, 1.66-1.88), 1.68 (95% CI, 1.60-1.77), and 1.67 (95% CI, 1.53-1.83), respectively. This tendency was consistently observed in analysis that simultaneously considered the number of comorbidities and individual disorder.

**Table 3.** Association Between Being Diagnosed With Schizophrenia and Psychiatric Comorbidities According to Several Studies

Comorbidities	Adjusted HRs (95% CI)	
	Model 1^a^	Model 2^b^
Multiple psychiatric comorbidities (reference, none)		
1 Comorbid disorder	2.03 (1.94-2.12)	1.91 (1.83-2.00)
2 Comorbid disorders	3.54 (3.35-3.75)	2.94 (2.77-3.13)
≥3 comorbid disorders	6.53 (6.05-7.04)	4.26 (3.90-4.65)
Individual psychiatric comorbidities		
Depression	1.82 (1.74-1.91)	1.68 (1.60-1.77)
Anxiety disorder	1.40 (1.32-1.48)	1.32 (1.25-1.39)
Tic disorder	2.05 (1.94-2.17)	1.77 (1.66-1.88)
Intellectual disability	1.91 (1.79-2.03)	1.83 (1.72-1.95)
Conduct disorder	1.27 (1.18-1.36)	1.21 (1.12-1.30)
ASD	2.72 (2.52-2.92)	2.43 (2.26-2.62)
Bipolar disorder	2.22 (2.05-2.40)	1.67 (1.53-1.83)
OCD	1.87 (1.69-2.07)	1.64 (1.47-1.82)
Nonorganic sleep disorder	1.57 (1.40-1.77)	1.40 (1.24-1.58)
SUD	1.22 (0.90-1.65)	1.11 (0.82-1.52)
Individual psychiatric comorbidities (reference, none)		
Depression	1.98 (1.85-2.12)	1.90 (1.77-2.03)
Anxiety disorder	1.31 (1.18-1.46)	1.28 (1.16-1.42)
Tic disorder	2.33 (2.15-2.52)	2.04 (1.88-2.21)
Intellectual disability	2.35 (2.16-2.56)	2.29 (2.11-2.49)
Conduct disorder	1.38 (1.23-1.56)	1.37 (1.22-1.54)
ASD	3.79 (3.41-4.21)	3.46 (3.11-3.84)
Bipolar disorder	1.67 (1.31-2.13)	1.57 (1.23-2.00)
OCD	2.92 (2.29-3.72)	2.46 (1.93-3.14)
Nonorganic sleep disorder	1.05 (0.72-1.52)	1.04 (0.72-1.50)
SUD	1.51 (0.57-4.02)	1.52 (0.57-4.05)
2 comorbid disorders	3.48 (3.29-3.69)	2.92 (2.75-3.11)
≥3 comorbid disorders	6.37 (5.91-6.88)	4.26 (3.90-4.65)

Abbreviations: ASD, autism spectrum disorder; HR, hazard ratio; OCD, obsessive-compulsive disorder; SUD, substance use disorder.

^a^ Model 1 adjusted sex, age, health insurance, and psychiatric hospitalization as covariates.

^b^ Model 2 adjusted sex, age, health insurance, psychiatric hospitalization, and psychotropic medication use as covariates.

In subgroup analysis according to age group, methylphenidate use, and the previous presence of bipolar disorder, ASD, and intellectual disability, we showed that the presence of psychiatric disorder was significantly associated with an increased risk of being diagnosed with schizophrenia; the magnitude of this association exhibited an increasing tendency with multiple comorbidities and a wide variety of individual comorbidity (eTable 4, eTable 5, and eTable 6 in Supplement 1). After 1:1 PS matching, we identified 70 590 patients in each group with well-balanced baseline characteristics (eTable 7 and eTable 8 in Supplement 1). Results of analyses using the PS-matched cohort also showed the largely consistent result with our main findings (eTable 9, eTable 10, eTable 11, and eTable 12 in Supplement 1).

Patients who did not have any psychiatric comorbidity still encountered various psychiatric comorbidities before being diagnosed with schizophrenia, as shown in Table 4. Notably, 3244 patients (73.8%) experienced at least 1 psychiatric comorbidity before schizophrenia onset, and this trend was consistently observed across all age groups (5-9 years, 1804 patients [71.8%]; 10-14 years, 1045 patients [77.2%]; 15-19 years, 395 patients [74.3%]). Although the specific comorbidities varied among different age groups, depression emerged as the most common incident psychiatric comorbidity, found in 2023 patients (46.0%), followed by anxiety disorders, found in 1326 patients (30.2%). The longitudinal association between ADHD and schizophrenia, including the occurrence of psychiatric comorbidities, is further illustrated in the Sankey diagram in the Figure; eFigure 2 in Supplement 1; and eTable 13, eTable 14, eTable 15, and eTable 16 in Supplement 2.

**Table 4.** Incidence of Psychiatric Comorbidities in the Trajectories From Attention-Deficit/Hyperactivity Disorder to Schizophrenia^a^

Comorbidities	Patients, No. (%)	Sum of follow-up duration, y	Incidence rate (per 100 person-years)
All patients (n = 4506)			
No incidence of psychiatric comorbidities	1152 (26.21)	2180.47	52.83
Incidence of psychiatric comorbidities	3244 (73.79)	NA	NA
Depression	2023 (46.02)	8212.54	24.63
Anxiety disorder	1326 (30.16)	5982.72	22.16
Tic disorder	885 (20.13)	3242.29	27.30
Intellectual disability	531 (12.08)	2420.55	21.94
Conduct disorder	737 (16.77)	3042.79	24.22
ASD	295 (6.71)	1253.52	23.53
Bipolar disorder	1090 (24.80)	4570.68	23.85
OCD	238 (5.41)	1108.09	21.48
SUD	55 (1.25)	293.62	18.73
Aged 5-9 y (n = 2583)			
No incidence of psychiatric comorbidities	707 (28.16)	1261.81	56.03
Incidence of psychiatric comorbidities	1804 (71.84)	NA	NA
Depression	1027 (40.90)	4213.79	24.37
Anxiety disorder	646 (25.73)	2876.00	22.46
Tic disorder	676 (26.92)	2487.79	27.17
Intellectual disability	313 (12.47)	1480.25	21.15
Conduct disorder	465 (18.52)	2025.13	22.96
ASD	201 (8.00)	835.35	24.06
Bipolar disorder	552 (21.98)	2398.58	23.01
OCD	103 (4.10)	533.12	19.32
SUD	12 (0.48)	69.00	17.39
Aged 10-14 y (n = 1380)			
No incidence of psychiatric comorbidities	308 (22.76)	711.49	43.29
Incidence of psychiatric comorbidities	1045 (77.24)	NA	NA
Depression	726 (53.66)	3007.07	24.14
Anxiety disorder	489 (36.14)	2366.80	20.66
Tic disorder	191 (14.12)	680.84	28.05
Intellectual disability	176 (13.01)	759.94	23.16
Conduct disorder	238 (17.59)	889.56	26.75
ASD	83 (6.13)	339.11	24.48
Bipolar disorder	407 (30.08)	1677.16	24.27
OCD	85 (6.28)	384.39	22.11
SUD	27 (2.00)	150.96	17.89
Aged 15-19 y (n = 543)			
No incidence of psychiatric comorbidities	137 (25.75)	207.17	66.13
Incidence of psychiatric comorbidities	395 (74.25)	NA	NA
Depression	286 (53.76)	996.77	28.69
Anxiety disorder	198 (37.22)	742.31	26.67
Tic disorder	27 (5.08)	76.44	35.32
Intellectual disability	47 (8.83)	181.54	25.89
Conduct disorder	40 (7.52)	129.92	30.79
ASD	19 (3.57)	81.39	23.34
Bipolar disorder	140 (26.32)	497.35	28.15
OCD	51 (9.59)	190.90	26.72
SUD	16 (3.01)	73.65	21.72

Abbreviations: ASD, autism spectrum disorder; NA, not applicable; OCD, obsessive-compulsive disorder; SUD, substance use disorder.

^a^ This analysis is conducted only in patients who received a schizophrenia diagnosis without psychiatric comorbidity at baseline.

**Figure.**
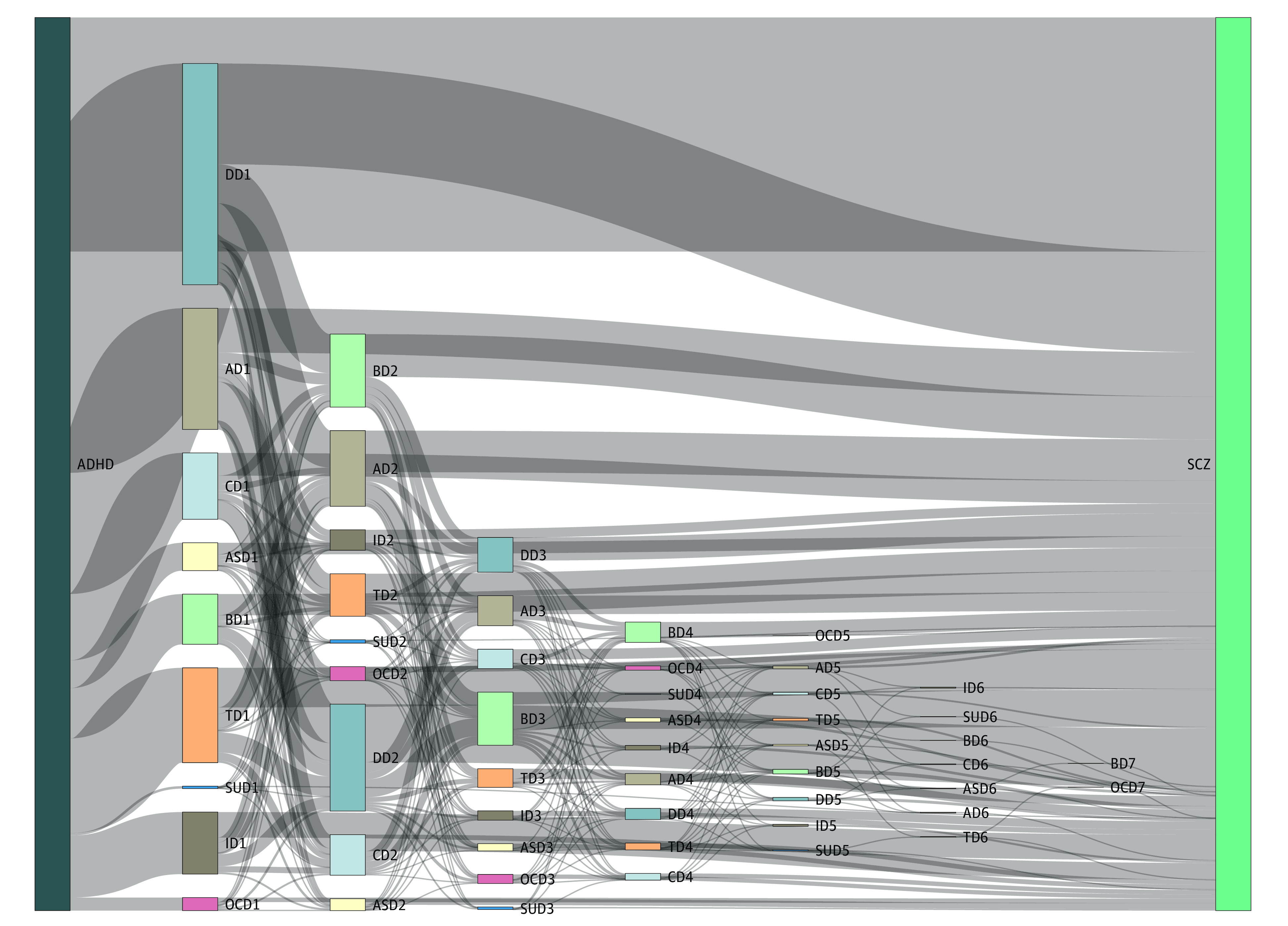
Sankey Diagram Illustrating the Incidence of Psychiatric Comorbidities in the Diagnostic Pattern From Attention-Deficit/Hyperactivity Disorder (ADHD) to Schizophrenia (SCZ) This analysis is specifically conducted in patients who received a schizophrenia diagnosis without any psychiatric comorbidity at baseline. The numbers for each disorder indicate the sequence of comorbidities that occured after the diagnosis of ADHD. AD indicates anxiety disorder; ASD, autism spectrum disorder; BD, bipolar disorder; CD, conduct disorder; DD, depression disorder; ID, intellectual disability; OCD, obsessive-compulsive disorder; SUD, substance use disorder; TD, tic disorder.

## Discussion

To our knowledge, this is the first study to investigate schizophrenia risk among children and adolescents with ADHD, with a particular focus on psychiatric comorbidities. Our findings suggested that patients with ADHD with psychiatric comorbidities had a higher risk of being diagnosed with schizophrenia than those without comorbidities. Furthermore, we observed a stepwise increase in schizophrenia risk with an increasing number of comorbidities. Regarding individual psychiatric comorbidities, ASD, intellectual disability, tic disorders, depression, and bipolar disorder showed relatively higher association with the increased schizophrenia risk in patients with ADHD. Additionally, to gain a better understanding of the progression toward schizophrenia in patients without psychiatric comorbidities at baseline, we constructed diagnosis trajectories.

Our results are consistent with previous studies that identified several psychiatric disorders as risk factors for schizophrenia development.^28^ For example, Maibing et al^28^ examined schizophrenia risk in pediatrics with a broad range of psychiatric disorders and reported an increased schizophrenia risk in short- and long-term periods. However, since this study primarily focused on the incidence rates of patients with overall psychiatric disorders, it did not address the impact of psychiatric comorbidities in patients with ADHD. Another study^2^ conducted in Taiwan used the Taiwan national claims database to estimate the risk of psychosis in patients with ADHD and reported approximately 2- to 3-fold higher risk in patients with other psychiatric disorders than those without other disorders. Nevertheless, they did not account for common psychiatric comorbidities, including depression and anxiety, which are frequently observed in patients with ADHD. Furthermore, as most studies focused on the risk factors at baseline, evidence regarding the diagnosis pattern from ADHD to schizophrenia is lacking. Therefore, our study contributes additional evidence regarding the association between several psychiatric comorbidities and subsequent schizophrenia development among children and adolescents with ADHD.

We observed that patients with ADHD with psychiatric comorbidities were older than those without psychiatric comorbidities. Provided that schizophrenia frequently occurs between 10 and 25 years in male patients and between 25 and 35 years in female patients,^29^ the higher schizophrenia incidence in patients with psychiatric comorbidities seems to be associated with age. However, various models using Cox PH models with age adjustment and subgroup analysis by age group suggested that the presence of psychiatric comorbidities was independently associated with schizophrenia development.

Regarding individual psychiatric comorbidities, ASD, intellectual disability, tic disorder, depression, and bipolar disorder were the top 5 comorbid disorders associated with an increased risk of schizophrenia. These findings are consistent with previous studies that demonstrated a potential positive association between schizophrenia risk and these disorders.^2,30,31^ For example, Jutla et al^2^ showed that ASD was a strong risk factor for psychotic symptoms.^30^ Having a diagnosis of tic disorders and intellectual disability indicated an increased risk of receiving a schizophrenia diagnosis. Yung et al^32^ conducted a study on predicting psychosis development and reported that a diagnosis of depression and anxiety served as a significant and strong predictor of psychosis. Furthermore, when examining the diagnosis trajectory among patients without psychiatric comorbidities at baseline, these disorders were prominently incident during the follow-up period. These consistent findings across different studies further support the association between these psychiatric comorbidities and increased schizophrenia risk.

However, interestingly, patients with ASD showed a high association of being diagnosed with schizophrenia, but the overall incidence of ASD was relatively low in trajectories analyses. This trend might be associated with the fact that ASD is typically identified early in development compared with other psychiatric disorders.^33^ Furthermore, in contrast to previous studies, we found no statistically significant association of SUD and schizophrenia, which might be attributed to the small sample size of patients with SUD.

Moreover, we examined other schizophrenia-associated factors in patients with ADHD. Male sex, older age, and Medicaid were robust factors associated with risk for the incidence of schizophrenia in this population, which was consistent with previous findings.^34,35,36^ Furthermore, we observed that antipsychotic medication use was significantly associated with increased schizophrenia risk. A few studies showed that antipsychotics have the potential to provoke schizophrenia.^37^ Meanwhile, these drugs may be prescribed for treating severe mental disorders regardless of schizophrenia or psychosis. For instance, the adjuvant uses of antipsychotics with antidepressants have been commonly used for treating resistant depression. The use of anticholinergic medications also showed a consistent association with the increased risk of schizophrenia. Given that anticholinergic medications were commonly used for adverse effects of antipsychotics (such as dystonia and parkinsonism),^38^ this finding might be associated with the use of antipsychotics. Therefore, further study is needed to investigate the association between anticholinergic use and schizophrenia in patients with ADHD.

### Limitations

Several limitations should be considered when interpreting these findings. First, since the diagnosis of ADHD, schizophrenia, and other psychiatric comorbidities were based on diagnosis codes, the possibility of underdiagnosis or overdiagnosis could not be ruled out. Some patients with ADHD have chosen the general health consultation (*ICD Z* code) due to the social stigma surrounding mental health problems.^39^ Furthermore, owing to using the claims database, we could not consider the severity or symptoms of ADHD. Instead, psychiatric hospitalization was used as an indirect proxy for assessing symptom severity. Second, despite considering several potential confounders, completely controlling all potential effects of confounders is challenging. However, when adjusting several confounders in Cox PH models and conducting several sensitivity analyses, we observed robust results regarding the associations between psychiatric comorbidities and being diagnosed with schizophrenia. Lastly, psychiatric comorbidities were assessed at baseline, and patients developing signs of these comorbidities during the follow-up period remained in the noncomorbidity group in the analysis. To address this limitation, we analyzed diagnosis trajectories to schizophrenia in patients without psychiatric comorbidities at baseline.

## Conclusions

This study suggests that the presence of psychiatric comorbidities was associated with an increased risk of being diagnosed with schizophrenia in children and adolescents with ADHD. Interestingly, although patients had no psychiatric comorbidities during ADHD diagnosis, the occurrence of these disorders was frequently observed before the schizophrenia diagnosis. These findings highlighted the significance of carefully monitoring psychiatric comorbidities in patients with ADHD to effectively mitigate the burden of schizophrenia.

## Notes

Supplement 1. **eTable 1.** ICD-10 Codes of ADHD and ATC Codes for ADHD Medications **eTable 2.** ICD-10 Codes for Psychiatric Comorbid Disorders **eTable 3.** ATC Codes for Other Psychotropic Medications **eFigure 1.** Flow Chart of Study Population Selection **eTable 4.** Association Between Transitioning to Schizophrenia and Psychiatric Comorbidities in Subgroup Analysis by Age Groups **eTable 5.** Association Between Transitioning to Schizophrenia and Psychiatric Comorbidities in Subgroup Analysis by the Use of Methylphenidate During Follow-Up Period **eTable 6.** Association Between Transitioning to Schizophrenia and Psychiatric Comorbidities in Subgroup Analysis of Patients Without Specific Psychiatric Disorder **eTable 7.** Distribution of Patients by the Presence of Psychiatric Comorbidities After Propensity Score Matching **eTable 8.** Demographics of ADHD Patients According to the Presence of Psychiatric Comorbidity **eTable 9.** Association Between Transitioning to Schizophrenia and Psychiatric Comorbidities in the PS Matched Cohort **eTable 10.** Association Between Transitioning to Schizophrenia and Psychiatric Comorbidities in Subgroup Analysis by Age Groups in the PS Matched Cohort **eTable 11.** Association Between Transitioning to Schizophrenia and Psychiatric Comorbidities in Subgroup Analysis by the Use of Methylphenidate During Follow-Up Period in the PS Matched Cohort **eTable 12.** Association Between Transitioning to Schizophrenia and Psychiatric Comorbidities in Subgroup Analysis of Patients Without Specific Psychiatric Disorder in the PS Matched Cohort **eFigure 2.** Sankey Diagram Illustrating the Incidence of Psychiatric Comorbidities in the Diagnostic Transition From ADHD to Schizophrenia in Patients According to Age Groups

Supplement 2. **eTable 13.** Proportion of Patients in Trajectories From ADHD to Schizophrenia in all Patients **eTable 14.** Proportion of Patients in Trajectories From ADHD to Schizophrenia, Aged 5 to 9 Years **eTable 15.** Proportion of Patients in Trajectories From ADHD to Schizophrenia, Aged 10 to 14 Years **eTable 16.** Proportion of Patients in Trajectories From ADHD to Schizophrenia, Aged 15 to 19 Years

Supplement 3.
Data Sharing Statement
