# Supplementary material for: Psychiatric Comorbidities and Schizophrenia in Youths With Attention-Deficit/Hyperactivity Disorder
Source: JAMA Netw Open. 2023 Nov 30;6(11):e2345793. doi: 10.1001/jamanetworkopen.2023.45793 (PMC10690465; doi:10.1001/jamanetworkopen.2023.45793)
Supplement: Supplement 1. — eTable 1. ICD-10 Codes of ADHD and ATC Codes for ADHD Medications eTable 2. ICD-10 Codes for Psychiatric Comorbid Disorders eTable 3. ATC Codes for Other Psychotropic Medications eFigure 1. Flow Chart of Study Population Selection eTable 4. Association Between Transitioning to Schizophrenia and Psychiatric Comorbidities in Subgroup Analysis by Age Groups eTable 5. Association Between Transitioning to Schizophrenia and Psychiatric Comorbidities in Subgroup Analysis by the Use of Methylphenidate During Follow-Up Period eTable 6. Association Between Transitioning to Schizophrenia and Psychiatric Comorbidities in Subgroup Analysis of Patients Without Specific Psychiatric Disorder eTable 7. Distribution of Patients by the Presence of Psychiatric Comorbidities After Propensity Score Matching eTable 8. Demographics of ADHD Patients According to the Presence of Psychiatric Comorbidity eTable 9. Association Between Transitioning to Schizophrenia and Psychiatric Comorbidities in the PS Matched Cohort eTable 10. Association Between Transitioning to Schizophrenia and Psychiatric Comorbidities in Subgroup Analysis by Age Groups in the PS Matched Cohort eTable 11. Association Between Transitioning to Schizophrenia and Psychiatric Comorbidities in Subgroup Analysis by the Use of Methylphenidate During Follow-Up Period in the PS Matched Cohort eTable 12. Association Between Transitioning to Schizophrenia and Psychiatric Comorbidities in Subgroup Analysis of Patients Without Specific Psychiatric Disorder in the PS Matched Cohort eFigure 2. Sankey Diagram Illustrating the Incidence of Psychiatric Comorbidities in the Diagnostic Transition From ADHD to Schizophrenia in Patients According to Age Groups [file jamanetwopen-e2345793-s001.pdf]

Supplemental Online Content

Jeon SM, Lee DY, Cha S, Kwon J-W. Psychiatric comorbidities and schizophrenia in youths with attention-deficit/hyperactivity disorder. *JAMA Netw Open*. 2023;6(11):e2345793. doi:10.1001/jamanetworkopen.2023.45793

eTable 1. ICD-10 Codes of ADHD and ATC Codes for ADHD Medications

eTable 2. ICD-10 Codes for Psychiatric Comorbid Disorders

eTable 3. ATC Codes for Other Psychotropic Medications

eFigure 1. Flow Chart of Study Population Selection

eTable 4. Association Between Transitioning to Schizophrenia and Psychiatric Comorbidities in Subgroup Analysis by Age Groups

eTable 5. Association Between Transitioning to Schizophrenia and Psychiatric Comorbidities in Subgroup Analysis by the Use of Methylphenidate During Follow-Up Period

eTable 6. Association Between Transitioning to Schizophrenia and Psychiatric Comorbidities in Subgroup Analysis of Patients Without Specific Psychiatric Disorder

eTable 7. Distribution of Patients by the Presence of Psychiatric Comorbidities After Propensity Score Matching

eTable 8. Demographics of ADHD Patients According to the Presence of Psychiatric Comorbidity

eTable 9. Association Between Transitioning to Schizophrenia and Psychiatric Comorbidities in the PS Matched Cohort

eTable 10. Association Between Transitioning to Schizophrenia and Psychiatric Comorbidities in Subgroup Analysis by Age Groups in the PS Matched Cohort

eTable 11. Association Between Transitioning to Schizophrenia and Psychiatric Comorbidities in Subgroup Analysis by the Use of Methylphenidate During Follow-Up Period in the PS Matched Cohort

eTable 12. Association Between Transitioning to Schizophrenia and Psychiatric Comorbidities in Subgroup Analysis of Patients Without Specific Psychiatric Disorder in the PS Matched Cohort

eFigure 2. Sankey Diagram Illustrating the Incidence of Psychiatric Comorbidities in the Diagnostic Transition From ADHD to Schizophrenia in Patients According to Age Groups

This supplemental material has been provided by the authors to give readers additional information about their work.

© 2023 Jeon SM et al. *JAMA Network Open.*

eTable 1. ICD-10 Codes of ADHD and ATC Codes for ADHD Medications

**Psychiatric disorder**	**ICD-10 code**
ADHD	F90
**ADHD medications (Stimulants and Non-stimulants)**	**ATC codes**
Methylphenidate<br>Modafinil, Atomoxetine, Clonidine	N06B

ADHD, attention deficit hyperactivity disorder; ICD-10, International Classification of Diseases

© 2023 Jeon SM et al. *JAMA Network Open.*

eTable 2. ICD-10 Codes for Psychiatric Comorbid Disorders [a]

Psychiatric disorder	ICD-10 code
Depression	F32, F33
Anxiety	F40, F41
Intellectual disability	F70–F79
Non-organic sleep disorder	F51
Tic disorder	F95
Bipolar disorder	F31
Autism spectrum disorder	F84
Conduct disorder	F91

ADHD, attention deficit hyperactivity disorder; ICD-10, International Classification of Diseases
a. We identified all subcodes within the ICD-10 code that corresponded to the presentation of the disease

eTable 3. ATC Codes for Other Psychotropic Medications

Classification	ATC code	Agents in the classification
Antipsychotics	N05A	
Typical antipsychotics		Chlorpromazine, Levomepromazine, Perphenazine, Haloperidol, Molindone, Chlorprothixene, Pimozide
Atypical antipsychotics		Blonanserin, Ziprasidone, Clozapine, Olanzapine, Quetiapine, Sulpiride, Amisulpride, Risperidone, Zotepine, Aripiprazole, Paliperidone
Antidepressants	N06A	Fluoxetine, Citalopram, Paroxetine, Sertraline, Fluvoxamine, Escitalopram, Venlafaxine, Milnacipran, Duloxetine, Desvenlafaxine, Clomipramine, Amitriptyline, Doxepin, Amoxapine, Moclobemide, Trazodone, Mirtazapine, Bupropion, Tianeptine
Anxiolytics	N05B	Diazepam, Chlordiazepoxide, Lorazepam, Bromazepam, Clobazam, Alprazolam, Ethyl loflazepate, Etizolam, Clotiazepam, Tofisopam, Hydroxyzine, and Buspirione
Anticholinergic drugs	N04A	Trihexyphenidyl, Biperiden, Procyclidine, and Benztropine
Antiepileptic drugs	N03A	Phenobarbital, Primidone, Phenytoin, Fosphenytoin, Ethosuximide, Clonazepam, Carbamazepine, Oxcarbazepine, Rufinamide, Valproic acid, Vigabatrin, Lamotrigine, Topiramate, Gabapentin, Levetiracetam, Zonisamide, Pregabalin, Stiripentol, and Lacosamide
Lithium	N05AN01	Lithium

© 2023 Jeon SM et al. *JAMA Network Open.*

eFigure 1. Flow Chart of Study Population Selection

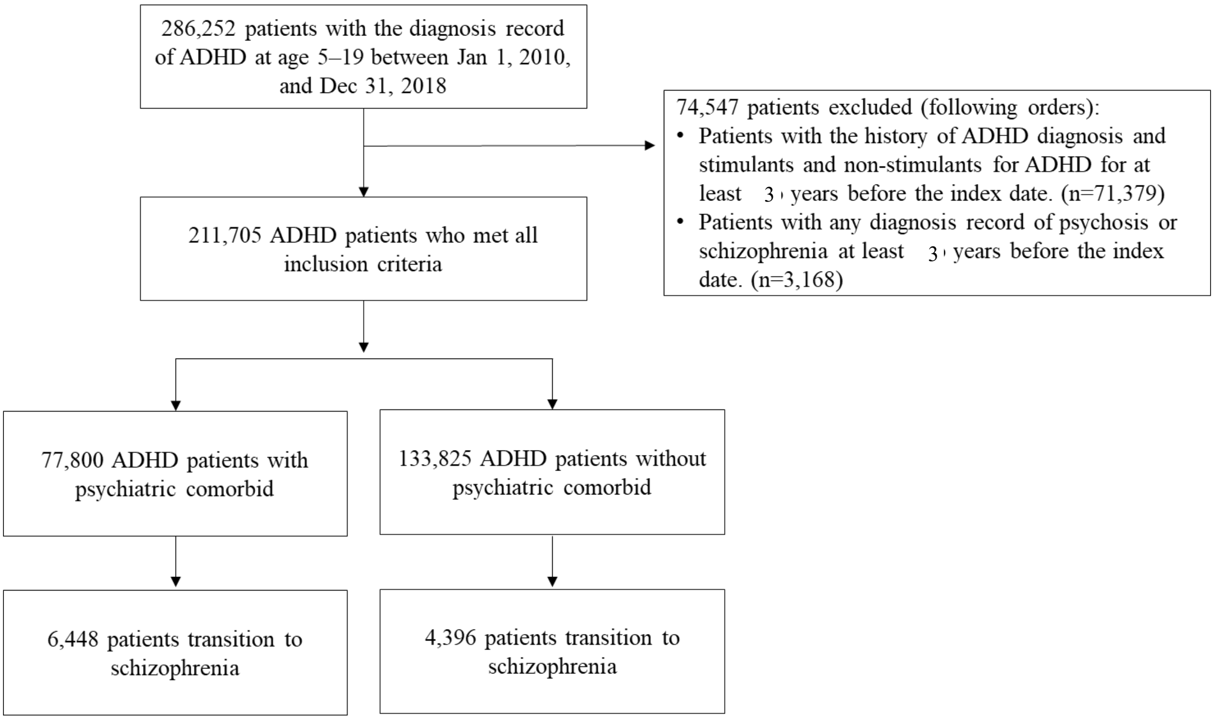

© 2023 Jeon SM et al. *JAMA Network Open.*

eTable 4. Association Between Transitioning to Schizophrenia and Psychiatric Comorbidities in Subgroup Analysis by Age Groups

	adjusted HR (95% CI) [a]		
	**Age 5-9**	**Age 10-14**	**Age 15-19**
**Presence of psychiatric comorbidities (Ref = none)**	2.11 (1.99-2.25)	2.15 (2.00-2.31)	2.20 (1.99-2.43)
**Multiple psychiatric comorbidities (Ref = none)**			
One comorbid disorder	1.91 (1.79-2.04)	1.89 (1.75-2.04)	1.94 (1.74-2.16)
Two comorbid disorders	3.02 (2.72-3.36)	3.07 (2.77-3.40)	2.71 (2.40-3.06)
Three or more comorbid disorders	4.71 (3.91-5.66)	4.45 (3.83-5.16)	3.78 (3.25-4.39)
**Individual psychiatric comorbidities**			
Depression (Ref=none)	1.76 (1.58-1.96)	1.67 (1.54-1.81)	1.59 (1.46-1.73)
Anxiety disorder (Ref=none)	1.23 (1.11-1.38)	1.28 (1.16-1.41)	1.37 (1.25-1.50)
Tic disorder (Ref=none)	2.02 (1.85-2.20)	1.77 (1.60-1.97)	1.39 (1.20-1.62)
Intellectual disability (Ref = none)	1.65 (1.50-1.82)	2.11 (1.90-2.35)	1.87 (1.64-2.14)
Conduct disorder (Ref = none)	1.34 (1.15-1.56)	1.34 (1.20-1.50)	1.02 (0.90-1.16)
ASD (Ref = none)	2.64 (2.38-2.93)	2.32 (2.00-2.69)	2.27 (1.92-2.69)
Bipolar disorder (Ref = none)	2.03 (1.67-2.47)	1.72 (1.48-2.01)	1.46 (1.28-1.67)
OCD (Ref=none)	1.54 (1.17-2.03)	1.59 (1.32-1.91)	1.69 (1.46-1.95)
Non-organic sleep disorder (Ref = none)	1.28 (0.88-1.85)	1.91 (1.52-2.40)	1.22 (1.05-1.43)
SUD (Ref = none)	2.40 (0.34-17.08)	0.90 (0.43-1.89)	1.20 (0.85-1.69)
**Number of comorbidities and individual psychiatric comorbidities (Ref = none)**			
Depression	1.75 (1.49-2.05)	1.81 (1.62-2.03)	2.03 (1.79-2.30)
Anxiety disorder	2.05 (1.84-2.27)	2.22 (1.93-2.55)	1.57 (1.30-1.89)
Tic disorder	1.07 (0.89-1.28)	1.28 (1.07-1.54)	1.98 (1.45-2.69)
Intellectual disability	1.95 (1.73-2.20)	2.71 (2.35-3.13)	2.83 (2.28-3.52)
Conduct disorder	1.44 (1.16-1.78)	1.34 (1.11-1.61)	1.42 (1.13-1.79)
ASD	3.44 (3.02-3.92)	3.34 (2.64-4.23)	4.31 (3.16-5.88)
Bipolar disorder	1.84 (1.11-3.06)	1.32 (0.85-2.06)	2.15 (1.48-3.12)
OCD	3.85 (2.45-6.04)	2.16 (1.35-3.44)	1.70 (1.18-2.43)
Non-organic sleep disorder	1.37 (0.81-2.32)	1.07 (0.51-2.26)	0.68 (0.32-1.44)
SUD	-	2.51 (0.63-10.06)	1.26 (0.31-5.03)
Two comorbid disorders	4.89 (4.07-5.87)	4.48 (3.85-5.20)	3.82 (3.29-4.44)
Three or more comorbid disorders	3.09 (2.78-3.43)	3.08 (2.78-3.41)	2.72 (2.41-3.07)

Attention deficit hyperactivity disorder, ADHD; hazard ratios, HRs; confidence intervals, CIs; autism spectrum disorder, ASD; obsessive-compulsive disorder, OCD; substance use disorder, SUD

a. The result was estimated using Model 2, adjusting sex, age, health insurance, psychiatric hospitalization, and psychotropic medication use as covariates.

© 2023 Jeon SM et al. *JAMA Network Open.*

eTable 5. Association Between Transitioning to Schizophrenia and Psychiatric Comorbidities in Subgroup Analysis by the Use of Methylphenidate During Follow-Up Period [a]

	adjusted HRs (95% CI) [a]	
	**Patients who used methylphenidate during follow-up period (n=142,493)**	**Patients who did not used methylphenidate during follow-up period (n=69,212)**
**Presence of psychiatric comorbidities (Ref = none)**	2.02 (1.92-2.12)	2.64 (2.43-2.87)
**Multiple psychiatric comorbidities (Ref = none)**		
One comorbid disorder	1.83 (1.74-1.93)	2.30 (2.11-2.52)
Two comorbid disorders	2.75 (2.55-2.96)	3.68 (3.29-4.11)
Three or more comorbid disorders	3.89 (3.48-4.34)	5.40 (4.65-6.27)
**Individual psychiatric comorbidities**		
Depression (Ref=none)	1.65 (1.55-1.76)	1.69 (1.54-1.86)
Anxiety disorder (Ref=none)	1.32 (1.23-1.41)	1.34 (1.22-1.47)
Tic disorder (Ref=none)	1.73 (1.59-1.89)	2.20 (2.00-2.41)
Intellectual disability (Ref = none)	1.80 (1.68-1.93)	1.75 (1.53-1.99)
Conduct disorder (Ref = none)	1.19 (1.09-1.30)	1.22 (1.06-1.40)
ASD (Ref = none)	2.37 (2.18-2.59)	2.41 (2.08-2.80)
Bipolar disorder (Ref = none)	1.59 (1.43-1.78)	1.79 (1.54-2.07)
OCD (Ref=none)	1.43 (1.25-1.64)	1.95 (1.66-2.29)
Non-organic sleep disorder (Ref = none)	1.44 (1.25-1.67)	1.40 (1.13-1.75)
SUD (Ref = none)	1.10 (0.75-1.62)	1.18 (0.71-1.98)
**Number of comorbidities and individual psychiatric comorbidities (Ref = none)**		
Depression	1.77 (1.63-1.92)	2.30 (2.00-2.65)
Anxiety disorder	1.27 (1.12-1.44)	1.43 (1.18-1.73)
Tic disorder	2.06 (1.84-2.30)	2.78 (2.45-3.14)
Intellectual disability	2.14 (1.95-2.35)	2.54 (2.11-3.06)
Conduct disorder	1.33 (1.16-1.52)	1.40 (1.08-1.80)
ASD	3.24 (2.88-3.65)	3.95 (3.13-4.98)
Bipolar disorder	1.92 (1.40-2.64)	4.01 (2.74-5.87)
OCD	1.44 (1.08-1.92)	2.03 (1.29-3.21)
Non-organic sleep disorder	1.04 (0.67-1.61)	1.22 (0.61-2.44)
SUD	2.37 (0.89-6.32)	-
Two comorbid disorders	2.72 (2.53-2.93)	5.44 (4.69-6.32)
Three or more comorbid disorders	3.87 (3.47-4.33)	3.67 (3.28-4.10)

Attention deficit hyperactivity disorder, ADHD; hazard ratios, HRs; confidence intervals, CIs; autism spectrum disorder, ASD; obsessive-compulsive disorder, OCD; substance use disorder, SUD

a. The result was estimated using Model 2, adjusting sex, age, health insurance, psychiatric hospitalization, and psychotropic medication use as covariates.

© 2023 Jeon SM et al. *JAMA Network Open.*

eTable 6. Association Between Transitioning to Schizophrenia and Psychiatric Comorbidities in Subgroup Analysis of Patients Without Specific Psychiatric Disorder [a]

	adjusted HRs (95% CI) [a]			
	**Patients without bipolar disorder (n=207,347)**	**Patients without ASD (n=204,838)**	**Patients without ID (n=199,265)**	**Patients without bipolar disorder, ASD, and ID (n=190,251)**
**Presence of psychiatric comorbidities (Ref = none)**	2.07 (1.98-2.16)	2.00 (1.91-2.09)	2.05 (1.96-2.15)	1.84 (1.76-1.93)
**Multiple psychiatric comorbidities (Ref = none)**				
One comorbid disorder	1.89 (1.81-1.98)	1.81 (1.73-1.89)	1.85 (1.77-1.94)	1.69 (1.78-1.61)
Two comorbid disorders	2.77 (2.60-2.95)	2.78 (2.61-2.97)	2.91 (2.72-3.11)	2.53 (2.73-2.35)
Three or more comorbid disorders	4.04 (3.64-4.49)	3.96 (3.59-4.36)	4.06 (3.67-4.49)	3.69 (4.21-3.23)
**Individual psychiatric comorbidities**				
Depression (Ref=none)	1.72 (1.63-1.82)	1.72 (1.63-1.81)	1.74 (1.65-1.84)	1.81 (1.71-1.92)
Anxiety disorder (Ref=none)	1.32 (1.25-1.40)	1.32 (1.25-1.40)	1.33 (1.25-1.41)	1.33 (1.24-1.41)
Tic disorder (Ref=none)	1.75 (1.64-1.87)	1.83 (1.72-1.95)	1.83 (1.72-1.95)	1.87 (1.75-2.01)
Intellectual disability (Ref = none)	1.85 (1.73-1.97)	2.02 (1.89-2.16)		
Conduct disorder (Ref = none)	1.23 (1.14-1.33)	1.22 (1.13-1.32)	1.25 (1.15-1.35)	1.28 (1.18-1.39)
ASD (Ref = none)	2.48 (2.29-2.68)		2.84 (2.61-3.09)	
Bipolar disorder (Ref = none)	-	1.72 (1.57-1.89)	1.73 (1.57-1.90)	
OCD (Ref=none)	1.69 (1.51-1.89)	1.69 (1.51-1.88)	1.68 (1.51-1.87)	1.80 (1.60-2.02)
Non-organic sleep disorder (Ref = none)	1.50 (1.31-1.72)	1.44 (1.27-1.63)	1.43 (1.26-1.62)	1.51 (1.31-1.75)
SUD (Ref = none)	1.20 (0.83-1.75)	1.10 (0.80-1.50)	1.13 (0.82-1.54)	1.19 (0.81-1.74)
**Number of comorbidities and individual psychiatric comorbidities (Ref = none)**				
Depression	1.87 (1.74-2.00)	1.88 (1.75-2.01)	1.91 (1.78-2.04)	1.86 (1.73-1.99)
Anxiety disorder	1.27 (1.15-1.41)	1.28 (1.15-1.42)	1.28 (1.16-1.42)	1.27 (1.14-1.41)
Tic disorder	2.03 (1.88-2.21)	2.06 (1.90-2.23)	2.05 (1.90-2.23)	2.06 (1.90-2.24)
Intellectual disability	2.28 (2.10-2.48)	2.29 (2.11-2.49)		
Conduct disorder	1.36 (1.21-1.53)	1.37 (1.22-1.54)	1.38 (1.23-1.55)	1.38 (1.22-1.55)
ASD	3.44 (3.09-3.82)		3.46 (3.11-3.84)	
Bipolar disorder		2.45 (1.92-3.13)	2.51 (1.96-3.20)	
OCD	1.54 (1.21-1.97)	1.55 (1.21-1.97)	1.58 (1.24-2.01)	1.53 (1.20-1.96)
Non-organic sleep disorder	1.03 (0.71-1.49)	1.03 (0.71-1.50)	1.04 (0.72-1.50)	1.03 (0.71-1.49)
SUD	1.51 (0.57-4.02)	1.51 (0.57-4.04)	1.54 (0.58-4.10)	1.53 (0.57-4.08)
Two comorbid disorders	2.74 (2.57-2.93)	3.97 (3.60-4.38)	4.11 (3.72-4.55)	2.56 (2.37-2.76)
Three or more comorbid disorders	4.01 (3.61-4.45)	2.78 (2.60-2.96)	2.90 (2.72-3.11)	3.76 (3.30-4.29)

Attention deficit hyperactivity disorder, ADHD; hazard ratios, HRs; confidence intervals, CIs; autism spectrum disorder, ASD; intellectual disability, ID; obsessive-compulsive disorder, OCD; substance use disorder, SUD
a. The result was estimated using Model 2, adjusting sex, age, health insurance, psychiatric hospitalization, and psychotropic medication use as covariates.

© 2023 Jeon SM et al. *JAMA Network Open.*

eTable 7. Distribution of Patients by the Presence of Psychiatric Comorbidities After Propensity Score Matching

	**Non matched cohort**			**Propensity score-matched cohort**		
	**ADHD with psychiatric comorbidity (n=77,880)**	**ADHD without psychiatric comorbidity (n=133,825)**	**aSD**	**ADHD with psychiatric comorbidity (n=70,590)**	**ADHD without psychiatric comorbidity (n=70,590)**	**aSD**
Sex_Female	23968 (30.8)	30465 (22.8)	0.182	20274 (28.7)	19666 (27.9)	0.019
Age (years)			0.525			0.046
5–9	31647 (40.6)	83434 (62.3)		31442 (44.5)	32041 (45.4)	
10–14	26309 (33.8)	38282 (28.6)		25780 (36.5)	26440 (37.5)	
15–19	19924 (25.6)	12109 (9.0)		13368 (18.9)	12109 (17.2)	
Health insurance_Medicaid	6244 (8.0)	8777 (6.6)	0.056	5069 (7.2)	4773 (6.8)	0.016
Healthcare utilization (Mean (SD))						
Overall utilization	18.87 (16.71)	17.04 (14.02)	0.119	18.28 (15.03)	18.21 (16.01)	0.005
Overall psychiatrist visits	0.06 (0.24)	0.05 (0.22)	0.056	0.06 (0.23)	0.05 (0.22)	0.018
Overall physicians visits	18.68 (16.66)	16.98 (14.01)	0.111	18.18 (15.04)	18.12 (16.00)	0.004
Inpatients psychiatric visits	243 (0.3)	20 (0.0)	0.074	130 (0.2)	12 (0.0)	0.05

Attention deficit hyperactivity disorder, ADHD; absolute standardized difference, Asd

© 2023 Jeon SM et al. *JAMA Network Open.*

eTable 8. Demographics of ADHD Patients According to the Presence of Psychiatric Comorbidity

	Total patients (n=141,180)	ADHD with psychiatric comorbidity (n=79,626)	ADHD without psychiatric comorbidity (n=79,626)	p-value
Transition to schizophrenia	8013 (5.68)	5549 (7.86)	2464 (3.49)	<.0001
Follow-up (years) (Median)	6.29	5.64	6.75	<.0001
Sex				
Male	101240 (71.71)	50316 (71.28)	50924 (72.14)	0.0003
Female	39940 (28.29)	20274 (28.72)	19666 (27.86)	
Age (years)				
5–9	63483 (44.97)	31442 (44.54)	32041 (45.39)	<.0001
10–14	52220 (36.99)	25780 (36.52)	26440 (37.46)	
15–19	25477 (18.05)	13368 (18.94)	12109 (17.15)	
Health insurance				
Health insurance	131338 (93.03)	65521 (92.82)	65817 (93.24)	0.002
Medicaid	9842 (6.97)	5069 (7.18)	4773 (6.76)	
Psychiatric hospitalization [a]	130 (0.09)	118 (0.17)	12 (0.02)	<.0001
Psychiatric comorbidities [a]				
The number of psychiatric comorbidities				
One comorbidity	52453 (37.15)	52453 (74.31)	-	
Two comorbidities	14423 (10.22)	14423 (20.43)	-	
Three or more comorbidities	3714 (2.63)	3714 (5.26)	-	
Individual psychiatric comorbid disorder [b]				
Depression	25441 (18.02)	25441 (36.04)	-	
Anxiety	16996 (12.04)	16996 (24.08)	-	
Tic disorder	14926 (10.57)	14926 (21.14)	-	
Intellectual disability	11706 (8.29)	11706 (16.58)	-	
Conduct disorder	9449 (6.69)	9449 (13.39)	-	
ASD	6563 (4.65)	6563 (9.30)	-	
Bipolar disorder	3462 (2.45)	3462 (4.90)	-	
Obsessive-Compulsive Disorder	2574 (1.82)	2574 (3.65)	-	
Non-organic sleep disorder	1911 (1.35)	1911 (2.71)	-	
Substance Use Disorder	220 (0.16)	220 (0.31)	-	
Use of psychotropic medications [a]				
Typical antipsychotics	1303 (0.92)	1256 (1.78)	47 (0.07)	<.0001
Atypical antipsychotics	7133 (5.05)	6909 (9.79)	224 (0.32)	<.0001
Anticholinergics	1915 (1.36)	1865 (2.64)	50 (0.07)	<.0001
Anxiolytics	30067 (21.30)	16635 (23.57)	13432 (19.03)	<.0001
SSRIs/SNRIs	11597 (8.21)	10311 (14.61)	1286 (1.82)	<.0001
TCAs	666 (0.47)	532 (0.75)	134 (0.19)	<.0001
MAOIs and others	1499 (1.06)	1320 (1.87)	179 (0.25)	<.0001
Antiepileptics	4913 (3.48)	3421 (4.85)	1492 (2.11)	<.0001
Lithium	422 (0.30)	386 (0.55)	36 (0.05)	<.0001

Attention deficit hyperactivity disorder, ADHD; autism spectrum disorder, ASD; selective serotonin reuptake inhibitors, SSRIs; serotonin and norepinephrine reuptake inhibitors, SNRIs; Tricyclic antidepressants, TCA; monoamine oxidase inhibitors, MAOIs

a. History of psychiatric hospitalization, psychiatric comorbidities, and the use of psychotropic medications were valued within one-year before the index date.

b. Patient with at least one diagnosis record of each disorder was considered to have each disorder.

© 2023 Jeon SM et al. *JAMA Network Open.*

eTable 9. Association Between Transitioning to Schizophrenia and Psychiatric Comorbidities in the PS Matched Cohort

	adjusted HR (95% CI) [a]
**Presence of psychiatric comorbidities**	2.10 (2.00-2.21)
**Multiple psychiatric comorbidities (Ref = none)**	
One comorbid disorder	1.86 (1.76-1.96)
Two comorbid disorders	2.93 (2.73-3.13)
Three or more comorbid disorders	4.36 (3.95-4.81)
**Individual psychiatric comorbidities**	
Depression (Ref=none)	1.67 (1.58-1.76)
Anxiety disorder (Ref=none)	1.28 (1.20-1.36)
Tic disorder (Ref=none)	1.80 (1.69-1.92)
Intellectual disability (Ref = none)	1.82 (1.70-1.94)
Conduct disorder (Ref = none)	1.23 (1.14-1.34)
ASD (Ref = none)	2.40 (2.22-2.60)
Bipolar disorder (Ref = none)	1.81 (1.64-1.99)
OCD (Ref=none)	1.69 (1.51-1.90)
Non-organic sleep disorder (Ref = none)	1.48 (1.29-1.71)
SUD (Ref = none)	1.24 (0.85-1.80)
**Number of comorbidities and individual psychiatric comorbidities (Ref = none)**	
Depression	1.83 (1.69-1.98)
Anxiety disorder	1.19 (1.06-1.34)
Tic disorder	2.03 (1.86-2.22)
Intellectual disability	2.27 (2.08-2.49)
Conduct disorder	1.36 (1.20-1.54)
ASD	3.38 (3.02-3.79)
Bipolar disorder	2.30 (1.73-3.04)
OCD	1.58 (1.21-2.05)
Non-organic sleep disorder	1.12 (0.77-1.64)
SUD	1.87 (0.70-4.98)
Two comorbid disorders	2.93 (2.74-3.14)
Three or more comorbid disorders	4.40 (3.98-4.85)

Attention deficit hyperactivity disorder, ADHD; hazard ratios, HRs; confidence intervals, CIs; autism spectrum disorder, ASD; obsessive-compulsive disorder, OCD; substance use disorder, SUD
a. The result was estimated using Model 2, adjusting sex, age, health insurance, psychiatric hospitalization, and psychotropic medication use as covariates.

© 2023 Jeon SM et al. *JAMA Network Open.*

eTable 10. Association Between Transitioning to Schizophrenia and Psychiatric Comorbidities in Subgroup Analysis by Age Groups in the PS Matched Cohort

	adjusted HR (95% CI) [a]		
	**Age 5-9**	**Age 10-14**	**Age 15-19**
**Presence of psychiatric comorbidities**	2.05 (1.90-2.22)	2.11 (1.95-2.29)	2.17 (1.95-2.41)
**Multiple psychiatric comorbidities (Ref = none)**			
One comorbid disorder	1.86 (1.72-2.02)	1.84 (1.69-2.01)	1.88 (1.68-2.11)
Two comorbid disorders	2.94 (2.61-3.30)	3.04 (2.73-3.39)	2.72 (2.37-3.12)
Three or more comorbid disorders	4.53 (3.73-5.50)	4.58 (3.92-5.36)	3.91 (3.28-4.67)
**Individual psychiatric comorbidities**			
Depression (Ref=none)	1.72 (1.54-1.92)	1.64 (1.51-1.79)	1.64 (1.48-1.81)
Anxiety disorder (Ref=none)	1.19 (1.07-1.33)	1.25 (1.13-1.38)	1.37 (1.22-1.54)
Tic disorder (Ref=none)	1.94 (1.77-2.13)	1.77 (1.59-1.97)	1.62 (1.35-1.94)
Intellectual disability (Ref = none)	1.60 (1.45-1.77)	2.10 (1.88-2.34)	1.95 (1.65-2.31)
Conduct disorder (Ref = none)	1.30 (1.12-1.52)	1.36 (1.21-1.52)	1.03 (0.88-1.21)
ASD (Ref = none)	2.55 (2.29-2.84)	2.36 (2.03-2.75)	2.24 (1.81-2.78)
Bipolar disorder (Ref = none)	2.06 (1.69-2.51)	1.81 (1.55-2.12)	1.61 (1.36-1.91)
OCD (Ref=none)	1.57 (1.20-2.07)	1.59 (1.32-1.91)	1.84 (1.54-2.20)
Non-organic sleep disorder (Ref = none)	1.23 (0.84-1.81)	2.02 (1.59-2.55)	1.24 (1.02-1.52)
SUD (Ref = none)	2.35 (0.33-16.70)	1.05 (0.50-2.21)	1.32 (0.84-2.07)
**Number of comorbidities and individual psychiatric comorbidities (Ref = none)**			
Depression	1.72 (1.45-2.02)	1.76 (1.57-1.98)	1.99 (1.73-2.28)
Anxiety disorder	1.04 (0.86-1.25)	1.24 (1.03-1.50)	1.37 (1.08-1.72)
Tic disorder	1.99 (1.77-2.23)	2.19 (1.89-2.53)	1.97 (1.38-2.82)
Intellectual disability	1.91 (1.68-2.17)	2.65 (2.28-3.07)	3.05 (2.38-3.91)
Conduct disorder	1.41 (1.13-1.75)	1.31 (1.09-1.59)	1.43 (1.09-1.86)
ASD	3.32 (2.89-3.81)	3.40 (2.68-4.32)	3.78 (2.58-5.54)
Bipolar disorder	3.73 (2.37-5.87)	2.01 (1.22-3.30)	1.75 (1.04-2.93)
OCD	1.81 (1.08-3.01)	1.36 (0.87-2.11)	1.73 (1.12-2.65)
Non-organic sleep disorder	1.35 (0.80-2.30)	1.08 (0.51-2.27)	0.82 (0.37-1.83)
SUD	-	2.50 (0.63-10.03)	1.75 (0.44-7.03)
Two comorbid disorders	3.00 (2.67-3.37)	3.04 (2.73-3.40)	2.72 (2.37-3.11)
Three or more comorbid disorders	4.70 (3.87-5.70)	4.63 (3.96-5.41)	3.92 (3.28-4.68)

Attention deficit hyperactivity disorder, ADHD; hazard ratios, HRs; confidence intervals, CIs; autism spectrum disorder, ASD; obsessive-compulsive disorder, OCD; substance use disorder, SUD

a. The result was estimated using Model 2, adjusting sex, age, health insurance, psychiatric hospitalization, and psychotropic medication use as covariates.

© 2023 Jeon SM et al. *JAMA Network Open.*

eTable 11. Association Between Transitioning to Schizophrenia and Psychiatric Comorbidities in Subgroup Analysis by the Use of Methylphenidate During Follow-Up Period in the PS Matched Cohort [a]

	adjusted HRs (95% CI) [a]	
	**Patients who used methylphenidate during follow-up period (n=93,929)**	**Patients who did not used methylphenidate during follow-up period (n=47,251)**
**Presence of psychiatric comorbidities**	2.00 (1.89-2.12)	2.49 (2.26-2.74)
**Multiple psychiatric comorbidities (Ref = none)**		
One comorbid disorder	1.80 (1.69-1.91)	2.12 (1.91-2.35)
Two comorbid disorders	2.74 (2.53-2.98)	3.54 (3.13-4.01)
Three or more comorbid disorders	4.02 (3.54-4.55)	5.35 (4.53-6.32)
**Individual psychiatric comorbidities**		
Depression (Ref=none)	1.65 (1.54-1.76)	1.65 (1.49-1.82)
Anxiety disorder (Ref=none)	1.28 (1.19-1.38)	1.31 (1.18-1.45)
Tic disorder (Ref=none)	1.77 (1.61-1.93)	2.23 (2.01-2.46)
Intellectual disability (Ref = none)	1.78 (1.64-1.92)	1.73 (1.50-2.00)
Conduct disorder (Ref = none)	1.21 (1.11-1.33)	1.24 (1.06-1.45)
ASD (Ref = none)	2.36 (2.15-2.59)	2.34 (2.00-2.75)
Bipolar disorder (Ref = none)	1.68 (1.48-1.90)	2.05 (1.74-2.40)
OCD (Ref=none)	1.50 (1.29-1.75)	2.00 (1.67-2.39)
Non-organic sleep disorder (Ref = none)	1.61 (1.36-1.90)	1.37 (1.05-1.78)
SUD (Ref = none)	1.29 (0.82-2.04)	1.25 (0.65-2.42)
**Number of comorbidities and individual psychiatric comorbidities (Ref = none)**		
Depression	1.73 (1.58-1.89)	2.06 (1.76-2.41)
Anxiety disorder	1.17 (1.02-1.34)	2.62 (2.29-3.01)
Tic disorder	2.07 (1.84-2.33)	1.31 (1.06-1.62)
Intellectual disability	2.13 (1.93-2.36)	2.39 (1.95-2.93)
Conduct disorder	1.32 (1.14-1.52)	1.33 (1.01-1.76)
ASD	3.23 (2.84-3.66)	3.55 (2.76-4.57)
Bipolar disorder	1.76 (1.21-2.54)	1.73 (1.02-2.94)
OCD	1.53 (1.13-2.08)	3.90 (2.51-6.05)
Non-organic sleep disorder	1.17 (0.75-1.82)	1.17 (0.55-2.46)
SUD	2.81 (1.05-7.49)	-
Two comorbid disorders	2.75 (2.53-2.99)	3.58 (3.16-4.05)
Three or more comorbid disorders	4.06 (3.58-4.60)	5.47 (4.63-6.46)

Attention deficit hyperactivity disorder, ADHD; hazard ratios, HRs; confidence intervals, CIs; autism spectrum disorder, ASD; obsessive-compulsive disorder, OCD; substance use disorder, SUD

a. The result was estimated using Model 2, adjusting sex, age, health insurance, psychiatric hospitalization, and psychotropic medication use as covariates.

© 2023 Jeon SM et al. *JAMA Network Open.*

eTable 12. Association Between Transitioning to Schizophrenia and Psychiatric Comorbidities in Subgroup Analysis of Patients Without Specific Psychiatric Disorder in the PS Matched Cohort [a]

	adjusted HRs (95% CI) [a]			
	**Patients without bipolar disorder (n=137,718)**	**Patients without ASD (n=134,617)**	**Patients without ID (n=129,474)**	**Patients without bipolar disorder, ASD, and ID (n=190,251)**
**Presence of psychiatric comorbidities**	2.04 (1.94-2.14)	1.98 (1.88-2.08)	2.02 (1.91-2.12)	1.82 (1.72-1.92)
**Multiple psychiatric comorbidities (Ref = none)**				
One comorbid disorder	1.85 (1.75-1.95)	1.76 (1.67-1.86)	1.79 (1.70-1.89)	1.65 (1.75-1.56)
Two comorbid disorders	2.75 (2.56-2.95)	2.79 (2.60-3.00)	2.93 (2.72-3.15)	2.57 (2.80-2.37)
Three or more comorbid disorders	4.13 (3.67-4.64)	4.04 (3.63-4.51)	4.15 (3.71-4.65)	3.72 (4.32-3.20)
**Individual psychiatric comorbidities**				
Depression (Ref=none)	1.69 (1.60-1.79)	1.70 (1.60-1.80)	1.74 (1.64-1.84)	1.80 (1.69-1.91)
Anxiety disorder (Ref=none)	1.28 (1.19-1.36)	1.28 (1.20-1.37)	1.28 (1.20-1.37)	1.28 (1.19-1.37)
Tic disorder (Ref=none)	1.79 (1.67-1.91)	1.87 (1.74-2.00)	1.87 (1.75-2.00)	1.92 (1.78-2.07)
Intellectual disability (Ref = none)	1.83 (1.71-1.96)	2.01 (1.87-2.16)		
Conduct disorder (Ref = none)	1.26 (1.16-1.38)	1.25 (1.15-1.36)	1.28 (1.17-1.39)	1.31 (1.20-1.44)
ASD (Ref = none)	2.46 (2.26-2.67)		2.79 (2.55-3.05)	
Bipolar disorder (Ref = none)		1.87 (1.68-2.08)	1.86 (1.67-2.06)	
OCD (Ref=none)	1.74 (1.54-1.97)	1.75 (1.55-1.97)	1.77 (1.57-1.99)	1.87 (1.64-2.13)
Non-organic sleep disorder (Ref = none)	1.56 (1.33-1.82)	1.48 (1.28-1.71)	1.48 (1.28-1.72)	1.57 (1.33-1.86)
SUD (Ref = none)	1.58 (1.05-2.38)	1.26 (0.87-1.84)	1.27 (0.87-1.85)	1.58 (1.05-2.39)
**Number of comorbidities and individual psychiatric comorbidities (Ref = none)**				
Depression	1.81 (1.68-1.96)	1.82 (1.68-1.96)	1.84 (1.70-1.98)	1.81 (1.67-1.95)
Anxiety disorder	1.19 (1.06-1.33)	1.19 (1.06-1.33)	2.04 (1.87-2.22)	1.18 (1.05-1.32)
Tic disorder	2.04 (1.87-2.22)	2.04 (1.87-2.23)	1.19 (1.06-1.33)	2.06 (1.89-2.25)
Intellectual disability	2.27 (2.08-2.49)	2.28 (2.08-2.49)		
Conduct disorder	1.36 (1.20-1.54)	1.36 (1.20-1.54)	3.36 (3.00-3.76)	1.37 (1.20-1.55)
ASD	3.38 (3.01-3.78)		1.59 (1.22-2.07)	
Bipolar disorder		2.28 (1.72-3.02)	2.33 (1.76-3.08)	
OCD	1.56 (1.20-2.04)	1.57 (1.20-2.04)	1.12 (0.77-1.64)	1.56 (1.20-2.03)
Non-organic sleep disorder	1.12 (0.77-1.64)	1.12 (0.77-1.64)	1.89 (0.71-5.04)	1.12 (0.76-1.63)
SUD	1.86 (0.70-4.97)	1.86 (0.70-4.96)	2.95 (2.74-3.18)	1.88 (0.70-5.01)
Two comorbid disorders	2.76 (2.56-2.96)	2.80 (2.60-3.01)	4.25 (3.79-4.75)	2.60 (2.40-2.83)
Three or more comorbid disorders	4.14 (3.69-4.66)	4.07 (3.65-4.54)	1.26 (1.07-1.47)	3.80 (3.27-4.41)

Attention deficit hyperactivity disorder, ADHD; hazard ratios, HRs; confidence intervals, CIs; autism spectrum disorder, ASD; intellectual disability, ID; obsessive-compulsive disorder, OCD; substance use disorder, SUD

a. The result was estimated using Model 2, adjusting sex, age, health insurance, psychiatric hospitalization, and psychotropic medication use as covariates.

© 2023 Jeon SM et al. *JAMA Network Open.*

eFigure 2. Sankey Diagram Illustrating the Incidence of Psychiatric Comorbidities in the Diagnostic Transition From ADHD to Schizophrenia in Patients According to Age Groups [a]

(a) age 5–9

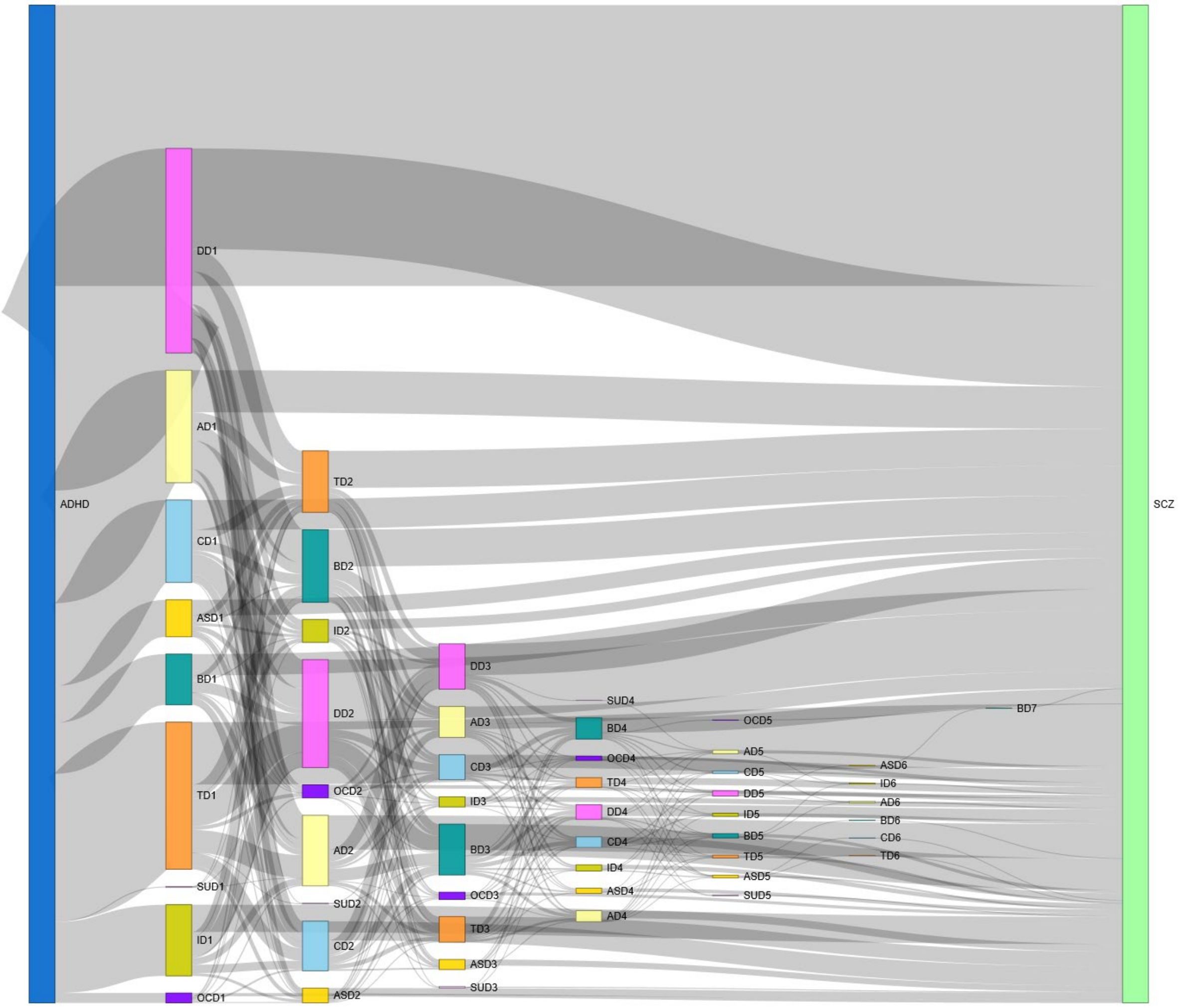

Attention deficit hyperactivity disorder, ADHD; depression disorder, DD; anxiety disorder, AD; conduct disorder, CD; bipolar disorder, BD; autism spectrum disorder, ASD; tic disorder, TD; substance use disorder, SUD, intellectual disability, ID; obsessive compulsive disorder, OCD. The numbers for each disorder indicate the sequence of comorbidities that occured after the diagnosis of ADHD

a. This analysis is specifically conducted in patients who transitioned to schizophrenia without any psychiatric comorbidity at baseline.

© 2023 Jeon SM et al. *JAMA Network Open.*

(Continued) eFigure 2.

(b) age 10–14 [a]

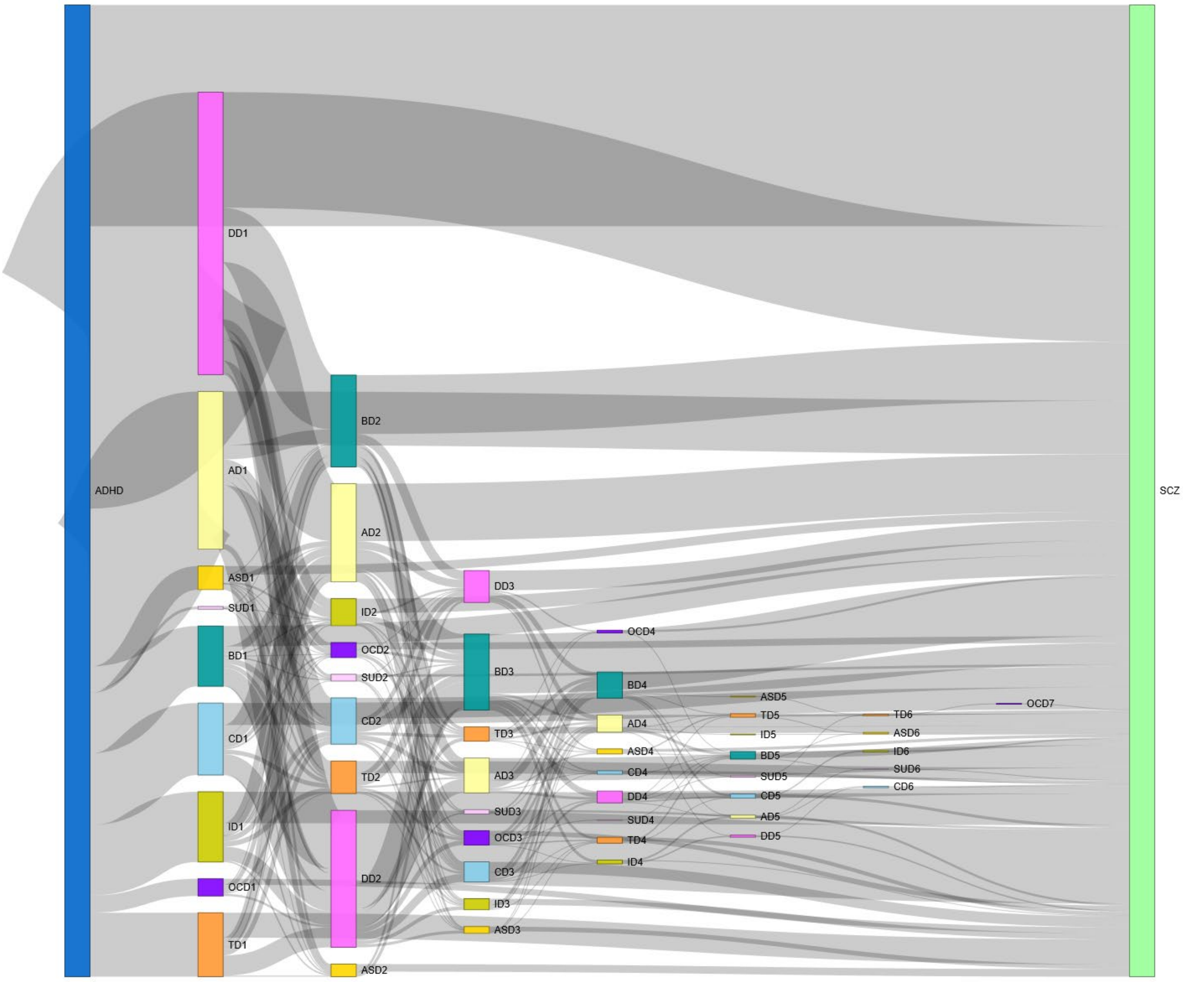

Attention deficit hyperactivity disorder, ADHD; depression disorder, DD; anxiety disorder, AD; conduct disorder, CD; bipolar disorder, BD; autism spectrum disorder, ASD; tic disorder, TD; substance use disorder, SUD, intellectual disability, ID; obsessive compulsive disorder, OCD. The numbers for each disorder indicate the sequence of comorbidities that occured after the diagnosis of ADHD

a. This analysis is specifically conducted in patients who transitioned to schizophrenia without any psychiatric comorbidity at baseline.

© 2023 Jeon SM et al. *JAMA Network Open.*

(Continued) eFigure 2.

(c) age 15–19[a]

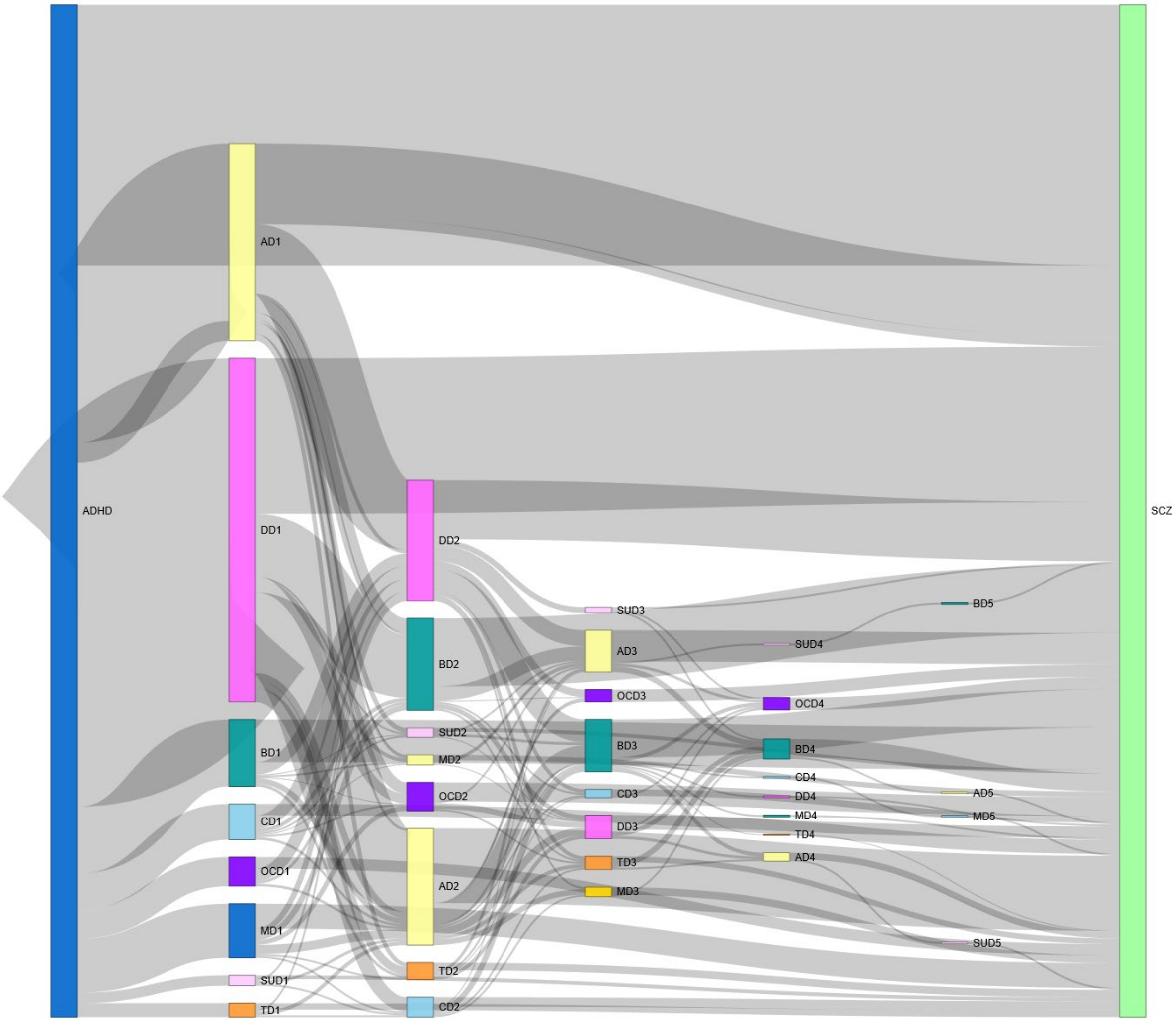

Attention deficit hyperactivity disorder, ADHD; depression disorder, DD; anxiety disorder, AD; conduct disorder, CD; bipolar disorder, BD; autism spectrum disorder, ASD; tic disorder, TD; substance use disorder, SUD, intellectual disability, ID; obsessive compulsive disorder, OCD. The numbers for each disorder indicated the sequence of comorbidities that occured after the diagnosis of ADHD

a. This analysis is specifically conducted in patients who transitioned to schizophrenia without any psychiatric comorbidity at baseline.

© 2023 Jeon SM et al. *JAMA Network Open.*
