# Supplementary material for: Psychiatric Comorbidities and Schizophrenia in Youths With Attention-Deficit/Hyperactivity Disorder
Source: JAMA Netw Open. 2023 Nov 30;6(11):e2345793. doi: 10.1001/jamanetworkopen.2023.45793 (PMC10690465; doi:10.1001/jamanetworkopen.2023.45793)
Supplement: Supplement 3. — Data Sharing Statement [file jamanetwopen-e2345793-s003.pdf]

Data Sharing Statement

Jeon. Psychiatric Comorbidities and Schizophrenia in Youths With Attention-Deficit/Hyperactivity Disorder. *JAMA Netw Open*. Published November 30, 2023. doi:10.1001/jamanetworkopen.2023.45793

Data
Data available: No

Additional Information
Explanation for why data not available: This study used Health Insurance Review and Assessment Service database (M20230801005). Requests to access these datasets should be directed to HIRA; Official website of HIRA: https://opendata.hira.or.kr; Contact information of data access committee: +82-33-739-1083
